# Long-term particulate matter exposure and mortality: a review of European epidemiological studies

**DOI:** 10.1186/1471-2458-9-453

**Published:** 2009-12-08

**Authors:** Claudio Pelucchi, Eva Negri, Silvano Gallus, Paolo Boffetta, Irene Tramacere, Carlo La Vecchia

**Affiliations:** 1 Istituto di Ricerche Farmacologiche "Mario Negri", Milan, Italy; 2 International Prevention Research Institute, Lyon, France; 3 Mount Sinai School of Medicine, New York, NY, USA; 4 Istituto di Statistica Medica e Biometria "G. A. Maccacaro", Università degli Studi di Milano, Milan, Italy

## Abstract

**Background:**

Several studies considered the relation between long-term exposure to particulate matter (PM) and total mortality, as well as mortality from cardiovascular and respiratory diseases. Our aim was to provide a comprehensive review of European epidemiological studies on the issue.

**Methods:**

We searched the Medline database for epidemiological studies on air pollution and health outcomes published between January 2002 and December 2007. We also examined the reference lists of individual papers and reviews. Two independent reviewers classified the studies according to type of air pollutant, duration of exposure and health outcome considered. Among European investigations that examined long-term PM exposure we found 4 cohort studies (considering total and cardiopulmonary mortality), 1 case-control study (considering mortality from myocardial infarction), and 4 ecologic studies (2 studies considering total and cardiopulmonary mortality and 2 studies focused on cardiovascular mortality).

**Results:**

Measurement indicators of PM exposure used in European studies, including PM10, PM2.5, total suspended particulate and black smoke, were heterogeneous. This notwithstanding, in all analytic studies total mortality was directly associated with long-term exposure to PM. The excesses in mortality were mainly due to cardiovascular and respiratory causes. Three out of 4 ecologic studies found significant direct associations between PM indexes and mortality.

**Conclusion:**

European studies on long-term exposure to PM indicate a direct association with mortality, particularly from cardiovascular and respiratory diseases.

## Background

Data from European epidemiological studies on the relation between long-term exposure to particulate matter (PM) and mortality are still limited, as most research on health effects of air pollution has focused on short-term exposure [1]. Besides information on the issue from a few US cohort studies, including the American Cancer Society Cancer Prevention Study II (ACS-CPS) [2], the Harvard Six Cities Study [3], and the Women's Health Initiative Observational Study [4], a few European studies provided new data during the last 5 years.

Ambient levels of several air pollutants are more variable within Europe than in the USA, and in some areas they are comparably high compared to US levels [1]. In a previous report, we reviewed the evidence of the association between long-term exposure to ambient PM and risk of lung cancer in Europe [5]. In this report, we provide a comprehensive review of European epidemiological studies considering the relation between long-term PM exposure and total mortality as well as mortality from cardiovascular and respiratory diseases. Studies conducted in North America and other areas of the world are mentioned in the Discussion whenever relevant for comparison purposes. The rationale for restricting analyses to Europe is based on differences in population density, exposure and characteristics of pollutants (e.g., due to a larger proportion of diesel vehicles as compared to North America or Japan), as well as in available measures of pollutants.

## Methods

This work is part of a wider project on air pollution and various health outcomes in Europe. We retrieved from PubMed the abstracts of all the journal articles on European epidemiologic studies included in the EPA report [1]. We identified the MeSH terms common to all the 96 papers, and defined a search string to select the scientific literature, as follows:

("air pollutants" [MeSH] OR "air pollution" [MeSH]) AND humans [MeSH] AND ("cardiovascular diseases" [MeSH] OR neoplasms [MeSH] OR "respiratory tract diseases" [MeSH] OR hospitalization [MeSH]).

We defined another search string to include papers not yet indexed for Medline (therefore without MeSH terms), as follows:

(air [tiab] OR pollut* [tiab] OR "particulate matter" [tiab] OR ambient [tiab]) AND (particulate* [tiab] OR PM [tiab] OR PM10 [tiab] OR PM2.5 [tiab] OR PM5 [tiab]) NOT (medline [sb]).

We used these strings to search the Medline database for papers published from January 2002 to December 2007. Furthermore, reference lists of a number of published papers (original articles and reviews) were examined. We retrieved 4497 papers, which were then included in an electronic database using Endnote (version 9). On the basis of title, abstract (when available), and keywords (MeSH terms), two independent reviewers independently classified all the papers, in order to identify studies of high interest, using a 5-point score. From the first selection, 3122 papers were eliminated (both reviewers' score <3). The other 1375 papers, over 100 of which were scored 5 by both reviewers, were further evaluated and classified - using a 10-point score - according to type of air pollutant, duration of exposure (acute, short- or long-term) and health outcome considered. From the second selection we eliminated 738 papers with both reviewers' score <5 (or one reviewer's score = 5 and the other's one <5). Among the other 637 papers, 186 were considered original European studies by both reviewers. Most of these studies evaluated the association with short-term exposure to ambient pollutants, including PM. For this review, we selected all European studies investigating long-term exposure to PM and total, cardiovascular and respiratory mortality, i.e., 3 cohort studies from the Netherlands, Germany and France, 1 record linkage study from Norway, 1 case-control study on myocardial infarction from Sweden, and 4 ecologic studies.

Ethical approval: This is a review paper which did not include research involving contact with human subjects, and followed the good research practice criteria of the proponent institutions.

## Analytic studies on total mortality

The major results of four European analytic studies that investigated long-term PM exposure and total mortality are described below and are summarized in Table 1.

**Table 1:** Analytical studies investigating the relation between long-term PM exposure and total mortality.

Reference, Study design, Country	Sex, age at enrolment	Number of deaths	Risk indicator, measurement unit	Relative risk (95% CI)
Hoek et al., 2002, NLCS cohort, the Netherlands	MF, 55-69	489	- Model 1^a^	- Model 1^a^
			BS, 10 μg/m^3^	1.04 (0.65-1.64)
			Living near a major road	1.53 (1.01-2.33)
			- Model 2^b^	- Model 2^b^
			BS, 10 μg/m^3^	1.31 (0.95-1.80)
Beelen et al., 2007, NLCS cohort, the Netherlands	MF, 55-69	17,674	- Model 3^c^	- Model 3^c^
			BS, 10 μg/m^3^	1.05 (1.00-1.10)
				
Filleul et al., 2005, PAARC cohort, France	MF, 25-59	2396	TSP, 10 μg/m^3 ^(24 areas)	1.00 (0.99-1.01)
			TSP, 10 μg/m^3 ^(18 areas)^d^	1.05 (1.02-1.08)
			BS, 10 μg/m^3 ^(24 areas)	0.99 (0.98-1.01)
			BS, 10 μg/m^3 ^(18 areas)^d^	1.07 (1.03-1.10)
				
Gehring et al., 2006, cohort, Germany	F, 50-59	399	Average 1-year concentration PM_10_, 7 μg/m^3^	1.08 (0.94-1.25)
			Average 5-year concentration PM_10_, 7 μg/m^3^	1.13 (0.99-1.30)
				
Naess et al., 2007a, cohort, Norway	M, 51-70	NA	PM_2.5_, quartile 4 vs 1	1.44 (1.32-1.58)
	F, 51-70	NA	PM_2.5_, quartile 4 vs 1	1.41 (1.27-1.43)
	M, 71-90	NA	PM_2.5_, quartile 4 vs 1	1.18 (1.10-1.26)
	F, 71-90	NA	PM_2.5_, quartile 4 vs 1	1.11 (1.05-1.17)

BS: black smoke; CI: confidence interval; NA: not available; NLCS: Netherlands Cohort Study; PAARC: Pollution Atmosphérique et Affections Respiratoires Chroniques; PM: particulate matter; TSP: total suspended particulate

^a ^Model 1: Local and regional effects were separately included in the model

^b ^Model 2: Local and regional effects were combined in a single variable

^c ^Model 3: Exposure was calculated according to local, urban and regional components

^d ^Six areas for which the NO/NO_2 _ratio was >3 were excluded

A cohort study conducted in the Netherlands [6] was based on a random sample of 5000 subjects selected from the over 120,000 participants to the Netherlands Cohort Study on Diet and Cancer (NLCS). All subjects were 55-69 years old at enrolment (in 1986), and were recruited from 204 of 714 municipalities with computerized population registries and covered by a cancer registry. Ninety percent of the sample (4492 subjects) was successfully followed up until 1994. Two different models were used to estimate relative risks (RR). In the first model, average regional background concentration of black smoke (BS) and distance of the residence from a major road were analyzed separately, while in the second model these two factors were combined by adding the background and the estimated contribution from living near a major road to air pollution concentrations. Subjects who had lived 10 years or longer at the same address near a major road had an increased risk of all cause mortality, the RR being 1.53 (95% confidence interval, CI, 1.01-2.33) after adjustment for several confounders. According to the second model, the RR for a 10 μg/m^3 ^increase in BS concentration was 1.31 (95% CI, 0.95-1.80). Additional analyses of the Netherlands study have been reported in an abstract form [7]. Using the case-cohort approach, the adjusted RR of mortality for all natural causes for a 10 μg/m^3 ^increase in BS was 1.03 (95% CI, 0.91-1.17). The corresponding RR in the full cohort analysis, including covariate information only on age, gender, active smoking and area-level socio-economic status, was 1.05 (95% CI, 1.00-1.10).

The Pollution Atmosphérique et Affections Respiratoires Chroniques (PAARC) cohort study [8] included 14,284 adults residing in 7 French cities, aged 25-59 years at enrolment in 1974 and followed-up until 2001 for vital status. The average exposures to BS and total suspended particulate (TSP) were estimated for the period 1974-76. Long-term exposure to BS (RR = 0.99) or TSP (RR = 1.00) did not affect the risk of mortality from all non-accidental causes. However, when the analyses were restricted to subjects from the 18 areas with a ratio of NO/NO_2 _<3 (i.e., excluding the 6 areas where the exposure measure was likely influenced by a busy roadside near the air monitor station), the RRs for total non-accidental mortality for a 10 μg/m^3 ^increase were 1.07 (95% CI, 1.03-1.10) for BS and 1.05 (95% CI, 1.02-1.08) for TSP.

In a German study conducted in various towns of North Rhine Westphalia [9], about 4800 women aged 50-59 years who participated in several cross-sectional surveys between 1985 and 1994 were followed-up until 2002-2003. The overall response rate to the surveys was 70%, and data on follow-up and address were obtained for 97% of the sample. A similar approach to the Dutch study was used, i.e., background exposure and distance of the residence from a road with >10,000 cars/day were estimated independently. Background exposure was computed as average concentrations for the year of recruitment and for the four years preceding recruitment. The adjusted RRs for all cause mortality for a 7 μg/m^3 ^increase in PM_10 _were 1.08 (95% CI, 0.94-1.25) for average exposure measured in the baseline year and 1.13 (95% CI, 0.99-1.30) when average exposure was measured on five years. Mutual adjustment of PM_10 _level and proximity to major roads did not materially change these estimates.

A record-linkage study was conducted on all inhabitants of Oslo aged 51-90 years in 1992, followed-up until 1998 (n = 143,842) [10]. Hazard ratios (HR) were calculated according to approximate quartiles of long-term PM_2.5 _and PM_10 _exposure, separately by sex and age at enrolment (51-70 and 71-90 years). Compared to those in the lowest exposure category, the HRs were 0.96 (95% CI, 0.89-1.04), 1.12 (95% CI, 1.03-1.22) and 1.48 (95% CI, 1.36-1.60) in subsequent quartiles of PM_2.5 _exposure for men aged 51-70 years. Corresponding estimates in older men were 0.99 (95% CI, 0.93-1.06), 1.10 (95% CI, 1.03-1.17) and 1.19 (95% CI, 1.12-1.27). Estimates in women were very similar to those of men of the corresponding age group. Adjustment for education and occupational class (HRs given in Table 1) did not materially modify the results.

## Analytic studies on mortality from cardiovascular and respiratory diseases

Besides total mortality, the cohort studies described above also presented data on mortality from cardiopulmonary diseases. Further, a Swedish case-control study provided results on the relation between long-term PM exposure and fatal (and non-fatal) myocardial infarction. A summary of European studies providing data on mortality from cardiovascular and respiratory diseases is presented in Table 2.

**Table 2:** Analytical studies investigating the relation between long-term PM exposure and cardiovascular and respiratory mortality.

Reference, Study design, Country	Sex, age at enrolment	Number of deaths by cause	Risk indicator, measurement unit	Relative risk (95% CI)
Hoek et al., 2002, NLCS cohort, the Netherlands	MF, 55-69	185 cardio-pulmonary	- Model 1^a^	- Model 1^a^
			BS, 10 μg/m^3^	1.34 (0.68-2.64)
			Living near a major road	1.95 (1.09-3.51)
			- Model 2^b^	- Model 2^b^
			BS, 10 μg/m^3^	1.71 (1.10-2.67)
Beelen et al., 2007, NLCS cohort, the Netherlands	MF, 55-69	7325 cardio-pulmonary	- Model 3^c^	- Model 3^c^
			BS, 10 μg/m^3^	1.06 (0.98-1.15)
		1046 respiratory	BS, 10 μg/m^3^	1.22 (0.99-1.49)
				
Filleul et al., 2005, PAARC cohort, France	MF, 25-59	546 cardio-pulmonary	TSP, 10 μg/m^3 ^(24 areas)	1.01 (0.99-1.03)
			TSP, 10 μg/m^3 ^(18 areas)^d^	1.06 (1.01-1.12)
			BS, 10 μg/m^3 ^(24 areas)	1.00 (0.97-1.02)
			BS, 10 μg/m^3 ^(18 areas)^d^	1.05 (0.98-1.12)
				
Gehring et al., 2006, cohort, Germany	F, 50-59	139 cardio-pulmonary	Average 1-year concentration PM_10_, 7 μg/m^3^	1.34 (1.06-1.71)
			Average 5-year concentration PM_10_, 7 μg/m^3^	1.59 (1.23-2.04)
				
Rosenlund et al., 2006, case-control, Sweden	MF, 45-70	272 myocardial infarction	All MI deaths PM_10_, 5 μg/m^3^	1.39 (0.94-2.07)
			In-hospital MI deaths PM_10_, 5 μg/m^3^	1.21 (0.75-1.94)
			Out of hospital MI deaths PM_10_, 5 μg/m^3^	1.84 (1.00-3.40)
				
Naess et al., 2007a, cohort, Norway		Cardio-vascular		
	M, 51-70	2007	PM_2.5_, quartile increase	1.10 (1.05-1.16)
	F, 51-70	946	PM_2.5_, quartile increase	1.14 (1.06-1.21)
	M, 71-90	4531	PM_2.5_, quartile increase	1.05 (1.01-1.08)
	F, 71-90	7480	PM_2.5_, quartile increase	1.03 (1.00-1.05)
		COPD		
	M, 51-70	233	PM_2.5_, quartile increase	1.27 (1.11-1.47)
	F, 51-70	203	PM_2.5_, quartile increase	1.09 (0.94-1.25)
	M, 71-90	503	PM_2.5_, quartile increase	1.10 (1.00-1.21)
	F, 71-90	516	PM_2.5_, quartile increase	1.05 (0.96-1.16)

BS: black smoke; CI: confidence interval; COPD: chronic obstructive pulmonary disease; MI: myocardial infarction; NLCS: Netherlands Cohort Study; PAARC: Pollution Atmosphérique et Affections Respiratoires Chroniques; PM: particulate matter; TSP: total suspended particulate

^a ^Model 1: Local and regional effects were separately included in the model

^b ^Model 2: Local and regional effects were combined in a single variable

^c ^Model 3: Exposure was calculated according to local, urban and regional components

^d ^Six areas for which the NO/NO_2 _ratio was >3 were excluded

In the Netherlands Cohort Study [6], most of the excess risk observed in total mortality appeared to be attributable to cardiopulmonary causes of death. The RR of cardiopulmonary mortality for subjects living near a major road was 1.95 (95% CI, 1.09-3.51). Updated analyses [7] showed RR near to unity for high traffic intensity on the nearest road using the case-cohort approach, and RRs of 1.06 (95% CI, 1.00-1.12) for cardiopulmonary mortality and 1.10 (95% CI, 0.95-1.26) for respiratory mortality in the whole cohort. With reference to BS exposure, the RRs for a 10 μg/m^3 ^increase were 1.04 (95% CI, 0.90-1.20) for cardiopulmonary and 1.16 (95% CI, 0.91-1.48) for respiratory mortality using the case-cohort approach. The corresponding RRs calculated on the whole cohort, adjusted for age, sex, smoking and area-level socio-economic status, were 1.06 (95% CI, 0.98-1.15) and 1.22 (95% CI, 0.99-1.49).

In the PAARC study [8], causes of death were available until 1998 for 96% of the sample. No association was found between cardiopulmonary mortality (International Classification of Diseases, ICD-9 codes: 401-440 or 460-519) and long-term exposure to BS (RR = 1.00) and TSP (RR = 1.01). When the analysis was restricted to 18 areas, the RRs for all cardiopulmonary causes were 1.06 (95% CI, 1.01-1.12) for a 10 μg/m^3 ^increase in TSP and 1.05 (95% CI, 0.98-1.12) for BS.

Similarly to the findings of the Netherlands Cohort Study, in the German study conducted in North Rhine Westphalia the effects of PM and traffic appeared stronger for - or limited to - cardiopulmonary mortality (ICD-9 codes: 400-440 or 460-519) [9]. The adjusted RRs for a 7 μg/m^3 ^increase in PM_10 _in the baseline year was 1.34 (95% CI, 1.06-1.71), while the RR was 1.59 (95% CI, 1.23-2.04) when exposure was measured for five years. Women living near a major road had a 70% (95% CI, 2%-181%) increased risk of dying from cardiopulmonary causes. Mutual adjustment of PM_10 _level and proximity to major roads did not materially change these estimates. Another analysis was conducted on the same data to investigate the impact of respiratory health on the association between PM_10 _exposure and cardiovascular mortality [11]. Findings from that study suggested that impaired respiratory health and long-term exposure to air pollution are independently associated with an increase in cardiovascular mortality.

A population-based case-control study on 1397 cases with first myocardial infarction and 1870 population controls resident in Stockholm county, aged 45-70 years, was conducted between 1992 and 1994 [12]. Response rates to the mailed questionnaire varied between 70% and 81% depending on sex and case-control status. For each subject, exposure to PM_10 _and PM_2.5 _was reconstructed from 1960 to a year prior to enrolment (1992-1994), i.e., for over 20 years, using data on traffic around the home address. Only data for long-term exposure to PM_10 _were used in the analysis, given the high correlation between PM_10 _and PM_2.5 _(r = 0.998). Logistic regression models adjusted for the matching variables, i.e. age, sex and hospital catchment area, smoking, physical inactivity, diabetes and socio-economic status were used to compute odds ratios (OR). Hypertension, body mass, job strain, diet, passive smoking, alcohol and coffee intake, and occupational exposure to motor exhaust and other combustion products were also evaluated, but did not appear to confound the relation with PM. With reference to fatal cases of myocardial infarction (n = 272), the OR was 1.39 (95% CI, 0.94-2.07) for an increase of 5 μg/m^3 ^in PM_10 _exposure. A borderline significant association was found when fatal cases were further restricted to 84 subjects who died out of hospital (OR = 1.84, 95% CI, 1.00-3.40), while the OR was 1.21 (95% CI, 0.75-1.94) for in-hospital deaths. Though this finding can be interpreted as supportive of an association between air pollution exposure and mortality, random variation in small subgroups cannot be excluded.

The Norwegian record-linkage study presented cause- and sex-specific HRs for PM_10 _and PM_2.5 _(and NO_2_) [10]. The crude HRs for cardiovascular mortality (ICD-9 codes: 390-459) for a quartile increase of PM_2.5 _were 1.11 (95% CI, 1.06-1.16) in 51-70 year old men, 1.06 (95% CI, 1.03-1.09) in 71-90 year old men, 1.16 (95% CI, 1.09-1.24) in 51-70 year old women, and 1.02 (95% CI, 1.00-1.05) in 71-90 year old women. With reference to mortality from chronic obstructive pulmonary disease (COPD, ICD-9 codes: 490-496), the corresponding HRs were 1.32 (95% CI, 1.17-1.49), 1.14 (95% CI, 1.04-1.24), 1.18 (95% CI, 1.03-1.34), and 1.09 (95% CI, 1.00-1.18). Further, the HRs for PM_2.5 _exposure adjusted for occupation and education (given in Table 2) and those for PM_10 _exposure were almost unchanged. In the same record-linkage investigation, another study considered the socio-economic correlates of PM exposure in subjects aged 50-74 years [13]. Overall, the risk of death from cardiovascular diseases (RR = 1.11, 95% CI, 1.07-1.15 for a quartile increase) and COPD (RR = 1.17, 95% CI, 1.09-1.25) was associated with PM_2.5 _exposure. When deprivation indicators were included in the models, however, the RRs became closer to unity. The authors concluded that more deprived neighbourhoods have higher levels of air pollution, and thus deprivation accounts for some of the excess mortality from several diseases, including cardiovascular and respiratory conditions, associated with air pollution in these neighborhoods.

## Ecologic studies

An Irish study compared mortality rates before and after the ban of coal sales in Dublin [14]. Given the deterioration of the air quality in the 1980s after a switch from oil to other fuels, mainly bituminous coal for domestic water and space heating, the Irish Government banned the marketing, sale and distribution of bituminous coals within the city of Dublin on September 1990. After the ban, the average BS concentrations declined by 35.6 μg/m^3 ^(70%). The authors compared age-adjusted death rates (directly standardized on the 1991 Irish census population) for the 72 months before (September 1984 to August 1990) and after (September 1990 to August 1996) the ban. Significant decreases were observed for all non-accidental and cardiovascular mortality in all seasons, and the decreases were more marked in winter. Significant decreases were observed for respiratory mortality overall, in winter and spring, while no significant change was observed overall for other causes. The authors concluded that control of PM could substantially diminish daily mortality. However, during the same period, mortality declined in several other European countries. Thus, a causal link between the decline in mortality and the ban of coal sales cannot be established. On the other hand, the authors interpreted the fact that the biggest declines were observed in winter, when the use of coal was highest, as supportive of their hypothesis.

Two studies compared air pollution levels in 1030 census enumeration districts in Sheffield, UK, to mortality and emergency hospital admission rates from coronary heart disease [15] and stroke [16]. For each district, a 5-year average PM_10 _concentration for the period 1994-1999 was computed. The districts were then divided according to quintiles of PM_10 _concentrations. The mean PM_10 _concentration in the highest quintile was 23.3 μg/m^3^, and 16.0 μg/m^3 ^in the lowest one. Mortality and emergency hospitalization rates were then computed for the districts' quintiles. For coronary heart disease, the rate ratios (adjusted for sex, age, deprivation and smoking prevalence) for the highest quintile compared to the lowest one were 1.08 (95% CI, 0.96-1.20) for mortality and 1.01 (95% CI, 0.90-1.14) for hospital admissions. Corresponding values for stroke were 1.33 (95% CI, 1.14-1.56) and 1.13 (95% CI, 0.99-1.29).

An ecologic study conducted in Great Britain [17], based on small residential areas, investigated the association between long-term BS and SO_2 _exposure and mortality overall and from selected causes, including cardiovascular and respiratory diseases. This study found a significant excess risk of total, cardiovascular, and particularly respiratory mortality. Stronger associations were reported between recent exposures (0-4 years) and respiratory mortality, with adjusted excess relative risk of 3.6% (95% CI, 2.6%-4.5%) for 10 μg/m^3 ^of BS.

## Discussion

In European analytic studies conducted between 2002 and 2007, mortality was associated with various measures of long-term exposure to PM. The excesses were mainly attributed to cardiovascular and respiratory causes.

Major North American studies on the issue found associations between fine particles and total mortality somewhat stronger than those of European investigations. In the Harvard Six Cities study [3], including 8096 white participants from various cities of the USA followed since the mid-1970 to 1998, the adjusted RRs for an increase of 10 μg/m^3 ^of PM_2.5 _were 1.16 (95% CI, 1.07-1.26) for total mortality, 1.28 (95% CI, 1.13-1.44) for cardiovascular diseases, and 1.08 (95% CI, 0.79-1.49) for respiratory causes. The Women's Health Initiative cohort study, including 65,893 post-menopausal women, found a RR for cardiovascular mortality of 1.76 (95% CI, 1.25-2.47) for an increase of 10 μg/m^3 ^of PM_2.5 _[4]. The corresponding RR for incidence of cardiovascular diseases was 1.24 (95% CI, 1.09-1.41). In the ACS-CPS-II study [2,18], that linked air pollution data with the individual data of approximately 500,000 adults from the USA, followed from 1982 to the end of 1998, the adjusted RRs for an increase of PM_2.5 _of 10 μg/m^3 ^were 1.06 (95% CI, 1.02-1.11) for all-cause mortality, 1.12 (95% CI, 1.08-1.15) for cardiovascular diseases plus diabetes, and 0.92 (95% CI, 0.86-0.98) for respiratory causes.

Efforts have been made to analyse the consistency of findings from European and North American studies, and to assess reasons for heterogeneity, also on the effect of short-term PM_10 _exposure on daily mortality [19]. The "Air Pollution and Health: A Combined European and North American Approach" (APHENA) study found comparably increased mortality risk estimates for European and US cities, whereas the acute effects of PM exposure were greater in Canadian cities. Some differences between geographic areas were noticed according to effect modification factors of the relation between PM_10 _and short-term mortality, including level of co-pollutants and climate (i.e., temperature and humidity).

In investigations of long-term effects of PM, the modifying effect of selected socio-economic factors varied across studies. In the USACS-CPS-II study, no association was found between PM_2.5 _and total and cardiopulmonary mortality for subjects in the highest category of education (>high school), while there was an association in less educated subjects [2]. This is in contrast with the findings from a French study [8], where the highest point estimates were observed in more educated subjects. Apart for chance and bias, at least part of the discrepancies could be explained by the different PM measure investigated, as most European studies did not provide data on PM_2.5_. Interestingly, an analysis of socio-economic correlates of PM exposure conducted in the Norwegian record-linkage study found that deprivation explained part of the excess mortality from cardiovascular and respiratory diseases associated with air pollution, as poor city areas experienced higher levels of air pollution. On the basis of these and other findings [2,8,13], one of the major issues to be addressed in future studies will be the role of socio-economic covariates (i.e., concurrent confounder and determinant of exposure) on the association between PM exposure and mortality.

With reference to morbidity from respiratory and cardiovascular diseases, European studies in adults do not provide consistent evidence of an association between PM exposure and chronic bronchitis or asthma, nor cardiovascular conditions [12,15,16,20-22]. Studies on PM_10 _and lung function, on the other hand, reported positive results [23,24].

The number of analytic European studies that investigated the long-term effects of PM exposure on mortality is limited. Furthermore, for all the studies, exposure assessment was estimated only on the home address, without considering the characteristics of the home (e.g., floor of residence) or other possible sources of exposure. The French study, however, excluded from the sample heads of households who were manual workers, to avoid confounding from other exposures, and occupation was controlled for in some studies. In short-term time series, comparisons are ideally made between different days within individuals, and adjustment for day of week and holidays is often performed. In contrast, studies on long-term exposure are necessarily based on comparisons between individuals, and on cumulative exposure assessment. Misclassification of exposure is likely to occur and to affect results. There are indications that exposure measurement errors affect risk estimates, in most instances leading to attenuated findings [1]. Measurement error might be lower for major urban areas, where more detailed information is generally available, and higher outside the urban setting [25]. Measurement indicators were heterogeneous across European studies, as several PM indexes were used, including PM_10_, PM_2.5_, BS and TSP. Their results may therefore be not comparable in various studies, particularly if fine or ultrafine particles have a major role. Among other potential limitations, spatial autocorrelation should be taken into account in the analyses of air pollutants and health, but only two studies considered the issue [8,25]. However, updated data from the NLCS investigation found no material difference in the original estimates when spatial autocorrelation in neighbourhood and municipality was accounted for [25]. On the other hand, ecologic studies are suggestive and hypothesis generating, but, given the potential bias inherent in their design, they cannot provide a solid base for inference.

## Conclusion

European studies on long-term exposure to PM indicate a direct association with mortality, particularly from cardiopulmonary diseases. Further studies relying on uniform methods to measure air particles, addressing the effects of socio-economic correlates of PM exposure, and considering the role of the chemical components of PM besides its mass [26] are however needed. Priority should be given to the inclusion of populations living in areas where PM is high (such as those from some regions of Central and Eastern Europe), among whom adverse health effects may be detected sooner than in less polluted areas of Europe.

## Competing interests

The authors declare that they have no competing interests.

## Authors' contributions

CP and SG wrote the manuscript. CLV and EN conceived and coordinated the study. PB gave important contribution for interpretation of data and writing of the manuscript. EN, SG and IT performed literature search and selected the papers of interest for this review. All authors revised the text and approved the final manuscript.

## Pre-publication history

The pre-publication history for this paper can be accessed here:

http://www.biomedcentral.com/1471-2458/9/453/prepub
